# Merohedral twins revisited: quinary twins and beyond

**DOI:** 10.1107/S2053273315018197

**Published:** 2016-01-01

**Authors:** Marianne Quiquandon, Denis Gratias, Abdullah Sirindil, Richard Portier

**Affiliations:** a Laboratoire de Métallurgie de l’UMR 8247, IRCP Chimie-ParisTech, 11 rue Pierre et Marie Curie, F-75005 Paris, France

**Keywords:** merohedral twins, quinary twins, Dürer structure, polygonal tilings

## Abstract

A twin is defined as being an external operation between two identical crystals that share a fraction of the atomic structure with no discontinuity from one crystal to the other. This includes merohedral twins, twins by reticular merohedry as well as coherent twins by contact where only the habit plane is shared by the two adjacent crystals (epitaxy). Interesting and original cases appear when the invariant substructure is built with positions belonging to the same -module as, for example, the quinary twin structure first drawn by Albrecht Dürer [(1525 ▸). *The Painter’s Manual: a Manual of Measurement of Lines, Areas and Solids by Means of Compass and Ruler*. Facsimile Edition (1977), translated with commentary by W. L. Strauss. New York: Abaris Books]. This paper will show that the Dürer twins, once defined in five-dimensional space, are simple merohedral twins, in the sense of Georges Friedel, leaving the five-dimensional lattice invariant. This analysis will be generalized to some other higher-order -modules.

## Abstract 2

The generalization of lattice invariance in merohedral twins to 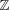-module invariance is discussed.

## 1. Introduction   

From an historical point of view, twins have played a special role in mineralogy and crystallography as they are an aggregate of identical crystals oriented with respect to each other in a very specific and characteristic manner (see, for instance, Groth, 1906 ▸; Putnis, 1992 ▸). Several twin laws have been proposed that can all finally be summarized by the basic idea that the specific relative orientation of a twinned crystal is a special isometry that keeps invariant – either exactly or approximately – a part of the atomic structure or of a specific property between the two twins. The idea is that the larger the common part is, the more stable is the twin and the more frequently it occurs in nature. Using this kind of intellectual guide, Friedel (1904 ▸, 1926 ▸, 1933 ▸) proposed a classification of twins based on the geometry of the sole crystal lattice (Bravais, 1851 ▸; Mallard, 1885 ▸; Donnay, 1940 ▸):

(i) Merohedral twins where the crystals share the same lattice (this can happen only for non-holohedral structures).

(ii) Twins by reticular merohedry where the crystals share only a fraction, a sublattice, of the crystal lattice; this corresponds, in metals and alloys, to the so-called special boundaries between grains like the famous mirror twin along the (111) direction often observed in the f.c.c. (face-centred cubic) metals.

(iii) Pseudo-merohedral twins or twins by reticular pseudo-merohedry where the previous definitions are satisfied only approximately.

In the present paper, we choose a general definition of twinning as being an operation between two identical crystals that share a fraction of the atomic structure or of a specific property with no discontinuity from one crystal to the other, in the spirit developed by Nespolo & Ferraris (2004 ▸), Grimmer & Nespolo (2006 ▸), Marzouki *et al.* (2014 ▸ and references therein):

(i) Twinned crystals in mutual orientation by reticular merohedry in three dimensions (two dimensions or one dimension) that share a common three-dimensional (two-dimensional or one-dimensional) sublattice.

(ii) A twin by contact where only the habit plane is shared by the two adjacent crystals (epitaxy).

(iii) More generally, any twin operation keeping invariant a fraction of the Wyckoff positions of the structure.

## 2. Formalism   

### 2.1. Symmetry operations and space groups   

**Figure 1:**
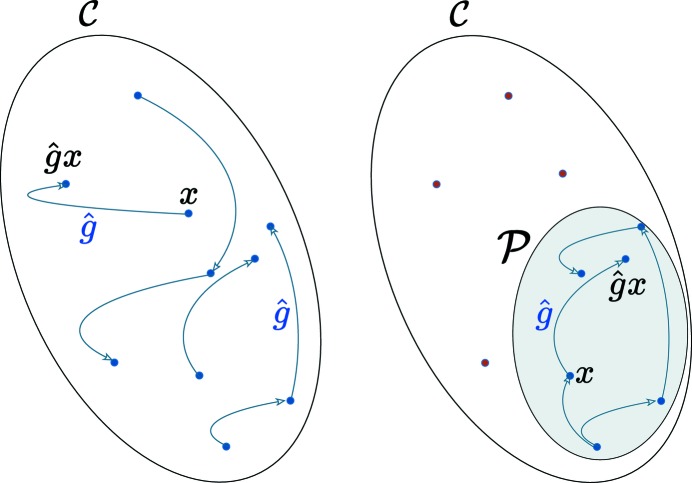
The space group 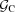 of a crystal is the set of all isometries that transform any point of the crystal into an equivalent one. The invariance group 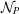 of a subset 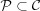 of the atoms of a crystal is generally different from 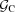 and is not necessarily a subgroup of 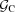.

A symmetry operation in 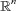 is an isometry of 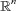, made of an orientation *g* and a translation part *t*, and noted 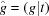, that transforms a point *r* in 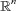 into a point 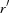 in 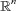 as 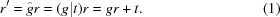
Designating by 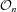 the set of the isometries of 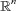, we define the space group 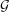 of the crystal as the set defined by
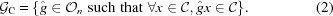
Considering now the subset 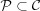 of the points that are invariant under the twin operation (see Fig. 1 ▸), its symmetry group 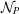 is defined 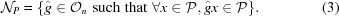
It is generally different to 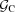 and is not necessarily a subgroup of it.

Thus, the fundamental group–subgroup relation defining the geometry of the two processes implies the intersection group 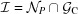, that gathers the symmetry operations that belong to both 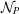 and 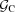.

**Figure 2:**
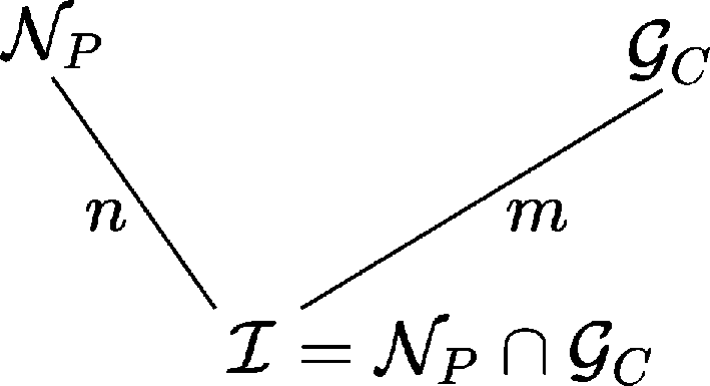
General group–subgroup tree characterizing a twin operation (see text).

The group scheme is shown in Fig. 2 ▸. It defines two integers *n* and *m* that are the indices of 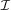 in, respectively, 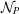 and 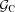: 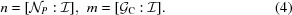
Their meaning is the following:

(i) 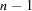 is the number of different possible twinned crystals around one given crystal and all share with the central crystal the same subset 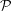 of symmetry group 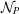: 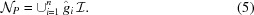



(ii) *m* is the number of equivalent subsets 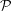 in the same crystal of space group 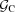: 
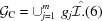
Each coset element 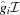 represents a twin operation,^1^ *i.e.* an operation that relates two twinned crystals sharing the same 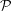 and each coset 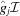 represents an internal operation for the crystal that transforms the subset 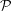 into one of its equivalents.

**Figure 3:**
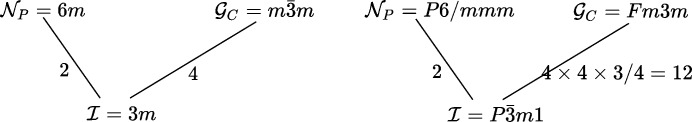
Two equivalent ways of describing the classical 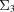 twin in f.c.c. metals. On the left, emphasis is put on the sublattice conservation between twinned crystals, whereas on the right, emphasis is put on the twinned crystals sharing a common 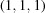 plane.

As a simple example, let us consider the standard so-called 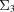 twin in cubic face-centred metals where the two individuals share a common sublattice of unit cell 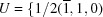, 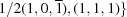. The involved groups are 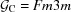, 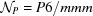 (unit cell *U*), with the intersection group 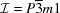 with the same unit cell *U*, as shown in Fig. 3 ▸ on the right. This leads to 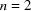, meaning that the twin operation connects two individuals and 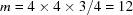 different crystals – corresponding to the 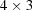 families of (1, 1, 1) planes, *A*, *B* or *C* – can be formed around one single crystal. Finally, the twin index that corresponds to the indices of the lattices only is 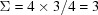, thus the term 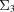 used to designate this kind of grain boundary.

This same twin can be as well defined through its epitaxy property and using point groups. The two adjacent crystals share the same (1, 1, 1) plane; thus, 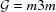, 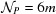 with an intersection group 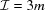. This means that the twin 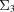, that keeps a (1, 1, 1) plane invariant, is between 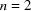 variants and 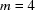 different individuals – the four orientation families of (1, 1, 1) planes – that can be formed around one given variant.

All known types of twins enter the general group–subgroup tree of Fig. 2 ▸.

For instance, merohedral twins are characterized by 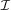 and 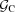 having the same lattice; coincidence grain boundaries are twins by reticular merohedry with a grain boundary index being the order of the lattice of 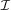 in the lattice of 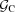.

## 3. Generalization   

**Figure 4:**
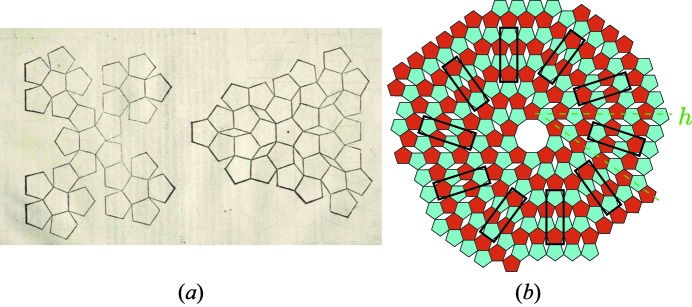
(*a*) Original drawings of pentagonal assemblages created by Dürer (1525 ▸) illustrating the fact that regular pentagons cannot tile the plane, one of the best packings being shown on the right. It is a two-dimensional periodic structure, designated here as the ‘Dürer structure’, multiply twinned around a central fivefold axis. (*b*) Construction by the authors of a tenfold twinning of the Dürer structure obtained by a spiral-like growing mode around a central empty decagon. The geometric nature of the twin interface *h* in green is shown in Fig. 6 ▸.

As we will show here, there are cases where the scheme of Fig. 2 ▸ leads to original results such as the twin structures first drawn by Albrecht Dürer and reproduced here in Fig. 4 ▸(*a*) from the original work *De symmetria partium in rectis formis humanorum corporum* (1532) and *Underweysung der Messung* (1538) (available on CD-ROM, Octavo Editions, CA, 2003).

### 3.1. The Dürer structure   

**Figure 5:**
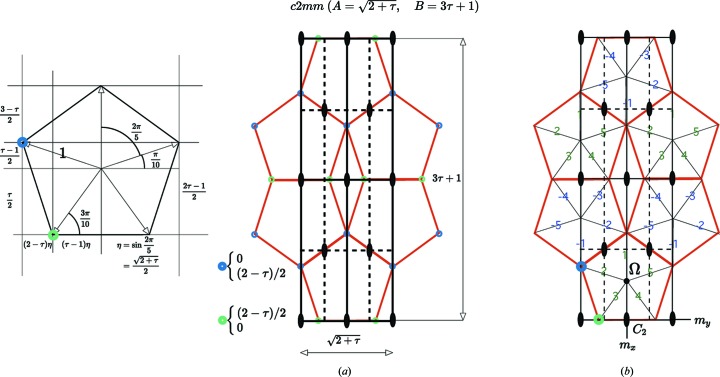
(*a*) Crystallographic description of the Dürer tiling. This very specific structure occurs almost perfectly as planar stacks of the Fe Wyckoff positions in the 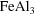 phase as identified by Black (1955*a* ▸,*b* ▸). It has been studied in detail by Ellner & Burkhardt (1993 ▸) and Ellner (1995 ▸) and has been taken as a basic example in the interpretation of twinning in icosahedral to cubic phase transformations in the (Al, Cu, Fe) system (Bendersky *et al.*, 1989 ▸; Bendersky & Cahn, 2006 ▸). (*b*) The Dürer structure can also be analysed as part of the 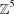-module built with the five vectors that relate the centre Ω to the five vertices of the elementary regular pentagon seen on the left. This periodic subset of the 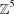-module has unit cell 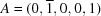 and 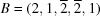 and two Wyckoff positions 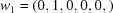 and 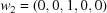.

The basic structure invented by Albrecht Dürer is shown in Fig. 5 ▸(*a*). It is built with six adjacent regular pentagons and has the crystallographic two-dimensional space group 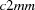. Taking the radius of the elementary pentagon as the unit length (see Fig. 5 ▸ *a* on the left), we find the lattice parameters 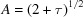 and 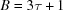 where τ is the golden mean 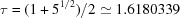
…. The whole structure is described by only two Wyckoff positions generated by the positions 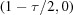 and 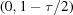 drawn in green and blue in Fig. 5 ▸.

Twins of the Dürer structure can be generated in a very symmetrical tenfold symmetry according to various equivalent modes, either radiant central as in Dürer’s original drawing, or spiral-like twins made of ten two-dimensional crystals along the ten directions of a regular decagon as seen in Fig. 4 ▸(*b*).

### 3.2. The hidden symmetries of the Dürer structure   

The very specific feature of the Dürer structure is that the atomic positions 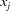 are all vertices of interconnected identical regular pentagons so that they can all be defined as *integer* sums of the five vectors relating the centre to the five vertices of the elementary pentagon: 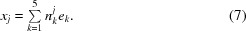
This structure is thus a periodic decoration of a 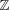-module^2^ of rank 5 (4 in fact, because the sum of the five unit vectors gives the zero vector) generated by the five vectors defined by the regular pentagon. In a more geometric view, the Dürer structure is a two-dimensional projection of a five-dimensional periodic structure in a four-dimensional hyperplane perpendicular to the five-dimensional main diagonal (1, 1, 1, 1, 1).

Embedding the Dürer structure in five-dimensional space is easily performed. We choose the origin in Ω as shown in Fig. 5 ▸(*b*) and find the unit cell defined by 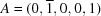 and 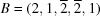, two vectors that are both perpendicular to the main diagonal 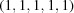 in five-dimensional space. The two Wyckoff positions are located at the nodes 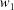 = 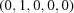 for the blue one and 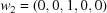 for the green one. The point symmetry operations are 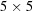 signed permutation matrices given by 
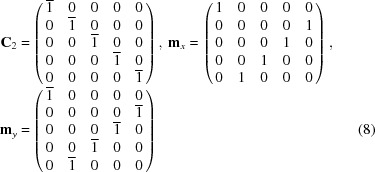
and the corresponding space operations that generate the space group 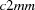 are
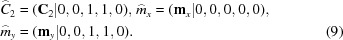
These operations together with the translation group generated by 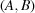 form a faithful representation of the group 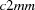 in five-dimensional Euclidean space.

### 3.3. Twin operation   

Now, we choose the underlying five-dimensional lattice generated by the five mutually orthogonal vectors 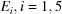 whose projections are the five basic vectors of the pentagon, as the geometric object that should be left invariant. The group 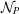 of all operations that keep the five-dimensional lattice invariant together with the two-dimensional cut space is 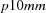 generated by 
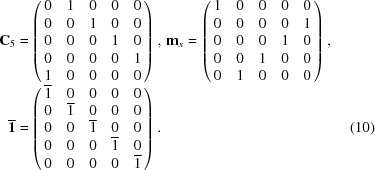
Thus, the general group–subgroup tree (Fig. 2 ▸) is built with the point groups 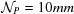 (order 20), 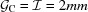 (order 4) that is a subgroup of 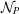 and thus qualifies this defect to be a * pure twin by merohedry* because 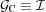, and thus it leaves the 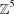-module invariant.

**Figure 6:**
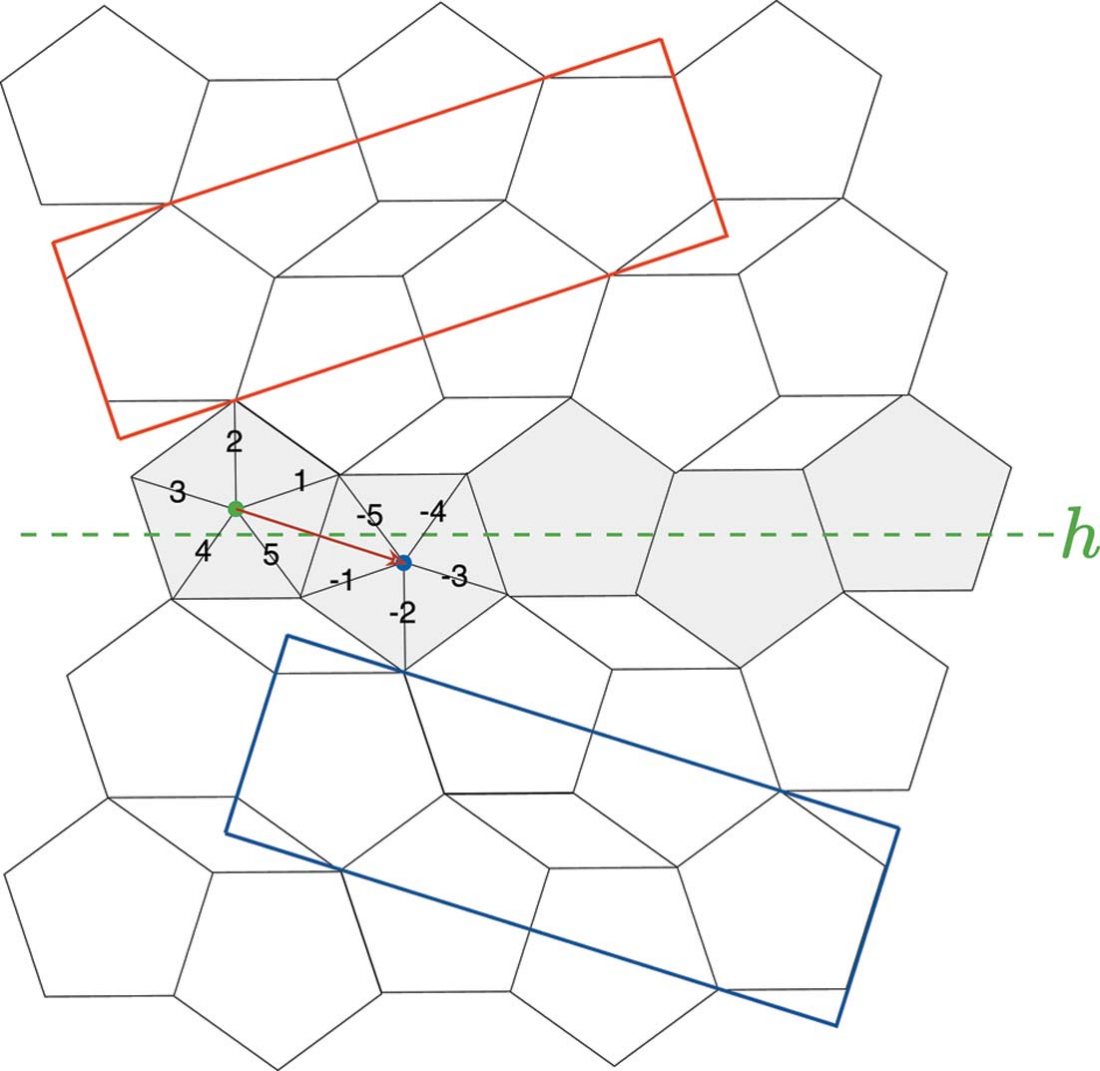
The twin operation of the Dürer structure is characterized by the horizontal glide mirror 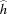 in green; it exchanges the green and the red unit cells and leaves invariant the common interface made of the collection of pentagons in grey that are shared by the two structures that are both built with the same pentagons.

The coset decomposition leads to five (20/4) different possible twins that are the five individuals drawn in Fig. 4 ▸(*b*). These are the five different ways of constructing the Dürer structure using the *same pentagon* (and its inverse). For example, one among the possible cosets of equivalent twin operations is given by
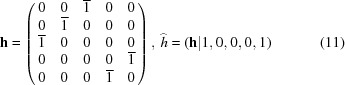
and is the glide mirror shown in Fig. 6 ▸.

It can be easily verified that this twin is *perfectly coherent* although it has no two-dimensional coincidence lattice. The boundary is defined by a sinuous row of adjacent pentagons that belong to both structures. Moreover, irrespective of the centring *c*, the lattice of the Dürer structure is the set of five-dimensional points 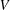 = 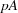 + 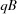 = 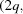 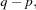 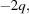 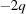, 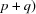 where *p* and *q* are integers. This lattice transforms into the set 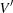 = 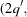 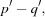 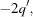 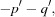 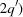 and the common lattice points are such that 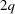 = 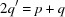 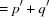 and 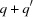 = 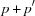 that has solutions only for 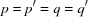, *i.e.* for the direction 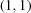. This is the habit direction of the twin: we have a perfect epitaxy with no (two-dimensional) coincident lattice.

## 4. Beyond the Dürer twin   

Dürer-like structures can easily be found using identical regular polygons of order *n* (later on, designated as *n*-gons) connected by edges. All these structures have the basic property of being defined by Wyckoff positions that are all on the same 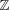-module and can thus be described as two-dimensional cut-and-projections of *n*-dimensional structures.

We discuss here some of the simplest of these kinds of polygonal tilings where the *n*-gons in the unit cell are all crystallographically equivalent. We shall designate these patterns as *monogeneous n*-gon patterns.

**Figure 7:**
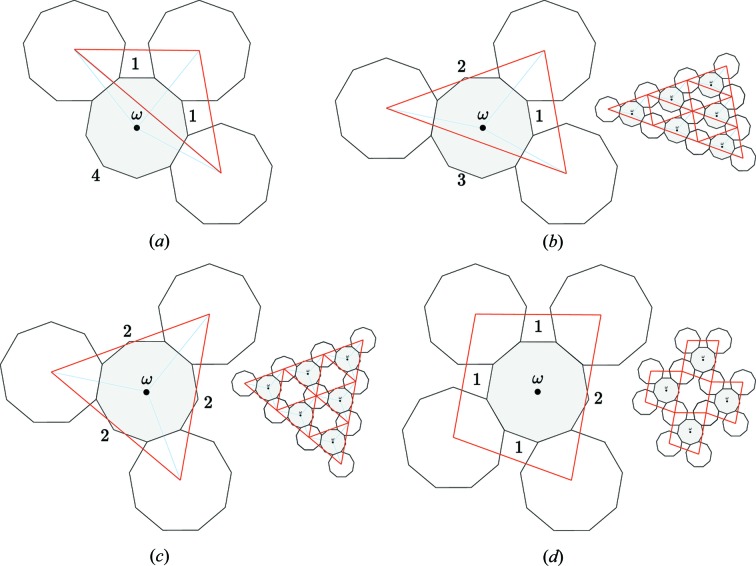
The local configurations of *n*-gons around a central one are characterized by the sequence of the number of free edges of the central *n*-gon that are between two consecutive connections. Here, for example, 9-gon tilings are shown with coordination 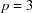 of configurations from (*a*) to (*c*): 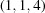, 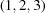 and 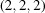. The configuration (*a*) 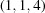 generates no periodic pattern of non-overlapping *n*-gons because the centre ω lies *outside* the triangle formed by the centres of the three adjacent *n*-gons. For the coordination 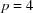, there is only one configuration 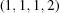 issued from (*a*) and shown in (*d*); but it leads to a non-monogeneous pattern since there are two kinds of *n*-gons, one (in grey) of coordination 4 and the other (in white) of coordination 2.

An efficient way of characterizing these patterns consists of reporting in a vector the sequence of the number of free edges between each connected edge around an *n*-gon as exemplified in Fig. 7 ▸ for 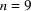. We call it the *vector of free edges*, the length of which is equal to the coordination of the *n*-gon. Under these notations, the Dürer structure of the previous section with 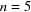 has coordination 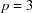 and is characterized, up to a circular permutation, by the vector 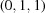.

The search for possible periodic solutions is significantly simplified by observing that, for an *n*-gon surrounded by *p* identical *n*-gons, the vector of free edges 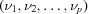 is such that 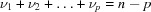 with 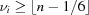.

Also, the maximum possible number 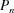 of non-overlapping *n*-gons sharing an edge of a central identical *n*-gon is given by 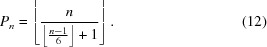



For the simple case 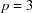, monogeneous non-overlapping *n*-gon periodic patterns are generated only if the centre of the central *n*-gon is *inside* the triangle formed by the centres of the three adjacent *n*-gons, in which case the triangle characterizes the unit cell of the structure (see Fig. 7 ▸).

The vector of free edges has the form 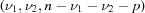. Assuming all index ranks are taken modulo *n*, the centres of the three *n*-gons are located at 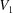 = (1, 1, 0, …), 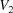 = (0, 0, …, 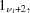 1, 0, …) and 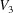 = (0, 0, …, 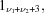 1, 0, …), generating the (primitive) unit cells defined by 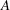 = 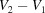 = 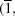 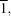 0, …, 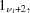 1, 0, …) and 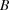 = 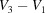 = 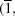 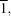 0, …, 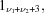 1, 0, …).

**Figure 8:**
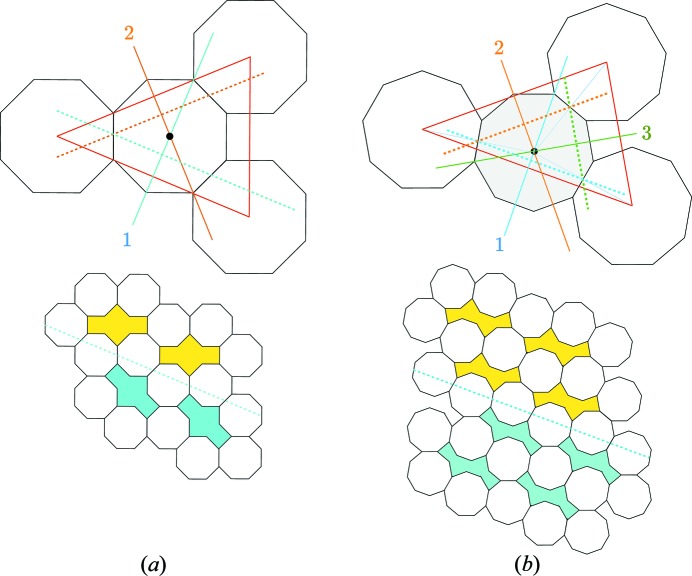
For the simple case of 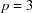, coherent twins are generated by exchanging two symbols in the vector of free edges while leaving the third one constant. Here is the example of the 8-gon 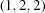 structure in (*a*) and 9-gon 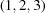 in (*b*). Because of its own symmetry the 8-gon structure allows for two exchanges only, 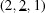, the mirror in blue, and 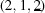, the mirror in orange. The 9-gon 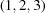 allows for one additional exchange 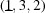 characterized by the mirror 3 in green. In all cases, the twin interfaces are generated by the glide mirrors drawn in dashed lines, perpendicular to the previous ones.

All twins in these structures are merohedral twins (built with the same *n*-gon). They are characterized by the symmetry elements of the *n*-dimensional lattice that leave the projected two-dimensional space invariant and that do not belong to the symmetry group of the structure as dictated by the general group tree of Fig. 2 ▸. In two-dimensional space, these twin operations are symmetry operations of the central *n*-gon that are not symmetry elements of the two-dimensional periodic structure and that leave invariant a row of the structure to form a perfect plane of epitaxy. For 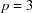, these elements are signed permutations of the *n* basic vectors generating the *n*-gon that transform into each other two of the other adjacent *n*-gons and put the third one in a new position. This translates in the vector of free edges in exchanging two symbols while keeping the third constant. For example, the vector of free edges 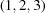 of the case 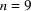 generates three possible coherent twinned crystals: 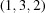, 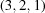 and 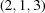 as exemplified in Fig. 7 ▸, whereas the vector of free edges 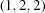 of the case 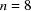 generates only two, 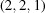 and 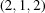. The interface operations are glide mirrors oriented along the common row of *n*-gons shared between the two adjacent crystals, as shown in Figs. 8 ▸ and 9 ▸.

## 5. Conclusion   

We propose here a formal extension of the notion of twin operation as an isometry between two identical crystals that preserves part of the atomic structure. Its internal symmetry group can possibly contain hidden symmetries issued from high-dimensional space when the Wyckoff positions of the preserved part of the structure belong to a 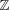-module of rank 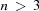. In that case, the concepts developed by Friedel survive very naturally by extending the notion of lattices to more general 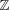-modules. Thus, twins that do not share a common sublattice can still be labelled as *merohedral* twins when they share the same 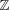-module as in the case of the Dürer twins.

Beyond this *n*-dimensional generalization, the interest of the present approach is the simplicity of its basic group–subgroup tree shown in Fig. 2 ▸ that can be used in all cases of actually known twins, once 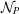 is identified.

## Figures and Tables

**Figure 9:**
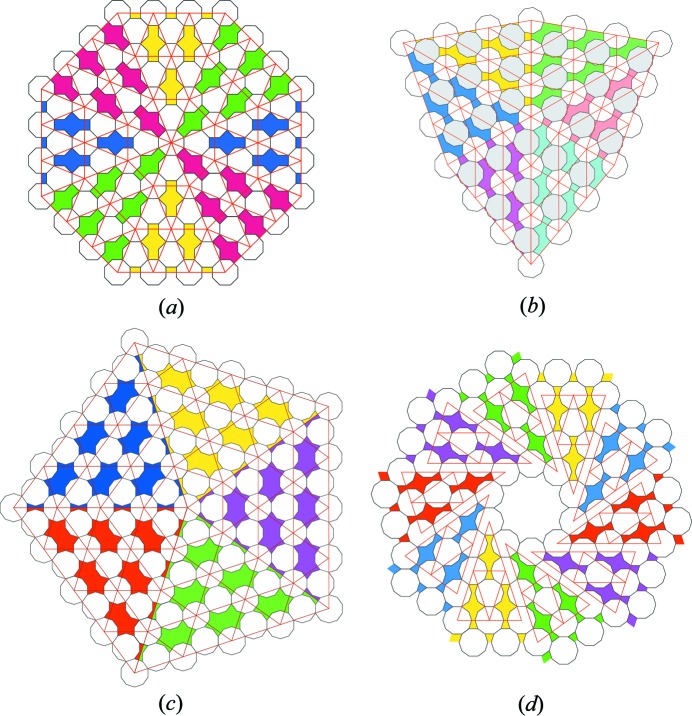
Examples of Dürer-like multiple twins for the (*a*) 8-gon 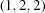, (*b*) 9-gon 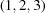, (*c*) 10-gon 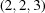 and (*d*) 10-gon 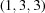 structures. In each drawing, the twinned crystals are built with the same *n*-gon.

## Footnotes

1 As discussed long ago (Guymont *et al.*, 1976 ▸; Gratias *et al.*, 1979 ▸; Portier & Gratias, 1982 ▸), interfaces (twins or grain boundaries) in homogeneous crystals are described by cosets of space groups. Indeed, consider two identical crystals related by the operation  and with space groups, respectively,  and . The operation  relates any point *x* of the first crystal to an equivalent  in the second crystal. Thus the twin operation is equivalently described by any operation that results in the product of any symmetry element of  by  and finally any symmetry element of , that is . This shows that a general interface operation between two identical crystals is not defined by a unique operation  but by a coset of the form .

2 A -module of rank *p* in  of dimension  is the set of points *x* defined by  where the basic vectors  are arithmetically independent (no non-zero *p* integers lead to a sum giving the null vector); it is isomorphic to an irrational projection in *d*-dimensional space of an *N*-dimensional lattice, with . For  the -module is a lattice.
